# Overexpression of LARP1 predicts poor prognosis of colorectal cancer and is expected to be a potential therapeutic target

**DOI:** 10.1007/s13277-016-5332-3

**Published:** 2016-09-10

**Authors:** Ling Ye, Sheng-tao Lin, Yu-shuai Mi, Yuan Liu, Yang Ma, Hui-min Sun, Zhi-hai Peng, Jun-wei Fan

**Affiliations:** 1 Department of General Surgery, Shanghai General Hospital, Shanghai Jiao Tong University School of Medicine, Shanghai, China; 2 Department of Pathology, Shanghai General Hospital, Shanghai Jiao Tong University School of Medicine, Shanghai, China

**Keywords:** Colorectal cancer, LARP1, Prognosis, Proliferation

## Abstract

This study investigated the significance of La-related protein 1 (LARP1) in the development and progression of colorectal cancer (CRC). Quantitative real-time polymerase chain reaction and Western blot analyses were carried out to determine the mRNA and protein expression of LARP1 in CRC tumor tissues and paired adjacent normal mucosa. The expression of LARP1 was upregulated in CRC. Immunohistochemical analysis using tissue microarray was performed. A positive correlation between LARP1 and proliferating cell nuclear antigen (PCNA) in the area of proliferation was observed using the Spearman’s correlation coefficient test (*r* = 0.332, *P* < 0.01). The elevated expression of LARP1 significantly correlated with T stage (*P* = 0.02), N stage (*P* = 0.006), M stage (*P* < 0.001), American Joint Committee on Cancer (AJCC) stage (*P* = 0.04), differentiation rank (*P* < 0.001), and PCNA level (*P* < 0.001). In addition, the inhibitory effect of LARP1 knockdown on CRC cell proliferation was demonstrated using Cell Counting Kit-8 (CCK8) and colony-forming cell (CFC) assays. Multivariate analysis showed that LARP1 was an independent prognostic factor for overall survival (OS; hazard rate (HR) = 0.244; 95 % confidence interval (CI), 0.078–0.769; *P* = 0.016) and disease-free survival (DFS; HR = 0.281; 95 % CI, 0.086–0.917; *P* = 0.035) in CRC patients. LARP1 plays an important role in the proliferation of colorectal cancer and represents a new prognostic indicator.

## Introduction

Colorectal cancer (CRC) is one of the most common cancers worldwide, with a high incidence and mortality [1]. In China, CRC is the fourth most common cancer and the fifth leading cause of death among all types of human cancer [2, 3]. Although CRC has a very high cure rate following surgical resection followed by multidisciplinary treatment (radiation and systemic chemotherapy) in the early stages, the high rate of relapse and distant metastasis still threaten a large percentage of patients [4, 5]. Efforts have been made to elucidate the genetic and molecular characteristics of CRC, for prognosis and response to targeted treatment [3, 5]. Tumor proteins such as P53, KRAS, and others have been associated with colorectal cancer progression and prognosis. However, tumor heterogeneity suggests the need for new markers of CRC [4, 6].

Candidate diagnostic markers in colon cancer were screened by combining experimental and bioinformatics approaches. Using laser capture microdissection, we obtained purified cell populations from heterogeneous tissues and found that La-related protein 1 (LARP1), an RNA-binding protein (RBP), was overexpressed in colorectal cancer tissues compared with paired normal specimens [7].

The LARP subfamilies comprising La, LARP1, LARP1b, LARP4, LARP4b, LARP6, and LARP7 play a significant role in transcription and/or mRNA translation [8, 9]. The members of the LARP family lack enzymatic domains but carry a La motif and a nearby RNA recognition motif with location varying among the different LARP proteins [8]. RBPs are important posttranscriptional regulators, with a key role in gene expression in a variety of human tumors [10–13]. LARPs are one of the RBPs that promote cell migration, invasion, and tumorigenesis in vivo [14]. Therefore, a comprehensive understanding of LARPs enables early detection of new biomarkers and facilitates the development of a strategy for cancer prevention and therapy.

LARP1 gene, which is located on chromosome 5q34, encodes a 1096-amino acid protein [15]. LARP1 carries a unique and highly conserved DM15 region, with unknown function [16]. However, DM15 has been suggested to mediate nucleic acid binding, which may be associated with oncogenesis [9, 17]. Recently, LARP1 was shown to be overexpressed in cervical and non-small cell lung cancers, with increased expression correlated with cancer progression and poor prognosis [14]. As a promising biomarker, the expression of LARP1 is closely related to poor prognosis of early-stage and alpha-fetoprotein-normal hepatocellular carcinoma (HCC) patients [18]. Recently, it has been found that downregulation of LARP1 expression in endometrial cancer and lung cancer cell lines was associated with decreased cell proliferation [19]. However, the role of LARP1 in the prognosis and cellular proliferation of colorectal cancer has yet to be defined.

Uncontrolled cell proliferation is considered to be an important feature of malignancy and is closely related to cancer progression. A previous study demonstrated that LARP1 is associated with the proliferation of endometrial cancer and lung cancer lines [19]. In this study, we explored the effect of LARP on the proliferation of colorectal cancer cells. As the accessory protein of DNA polymerase δ, Proliferating cell nuclear antigen (PCNA) plays an important role in cell proliferation, DNA replication, and repair [20]. In addition, PCNA also interacts with proteins involved in cell cycle regulation and checkpoint control [21]. The crucial role of PCNA in cellular proliferation supports its frequent use as a marker of tumor cell proliferation [22, 23]. Therefore, we combined PCNA with LARP1 to examine the effect of LARP1 on CRC cell proliferation.

This study initially evaluated the expression of LARP1 in CRC tissues and adjacent normal specimens to investigate the significance of LARP1 in the development and progression of CRC. Furthermore, our study determined the expression of LARP1 and PCNA using immunohistochemical analysis with CRC tissue microarrays (TMAs). We investigated the relationship with clinic pathological features as well as patient survival to illustrate the possibility of LARP1 as a prognostic marker in CRC. The correlation between LARP1 and PCNA in cancer cell proliferation was determined using the Spearman’s correlation coefficient test. The influence of LARP1 on CRC cell proliferation was studied after silencing the expression of LARP1 using short hairpin RNA (shRNA).

## Materials and methods

### Patients and tissue samples

Tissue samples were obtained from patients who had undergone radical resection at the General Surgery Department of Shanghai General Hospital. No chemotherapy or radiation therapy was applied to these patients before radical resection. We collected a total of 40 cases of colorectal cancer specimens between December 2014 and September 2015. The diagnosis was confirmed by two pathologists, and the cancer staging was determined based on pathological findings according to the American Joint Committee on Cancer (AJCC). All the CRC and corresponding adjacent normal mucosa specimens were collected and preserved after the approval of Institutional Research Ethics Committee of Shanghai Jiao Tong University Affiliated Shanghai General Hospital. All of the 40 patients in the study signed the informed consent. The CRC tissues and adjacent normal mucosae (at least 10 cm away from the primary site) were obtained after surgical resection and immediately frozen in liquid nitrogen and stored at −80 °C to extract RNA and protein later.

### RNA extraction and quantitative real-time polymerase chain reaction

The total RNA was extracted from cultured cells, tumor tissues, and paired normal mucosa from 40 colorectal cancer patients according to protocols of the manufacturer (TRIzol, TaKaRa, Japan). The first-strand cDNA was synthesized using 1 μg of total RNA by a RevertAid First Strand cDNA Synthesis Kit (Fermentas, Lithuania). Reverse transcription-polymerase chain reaction (RT-PCR) was performed using a ViiA 7 Real-Time PCR System (Life Technology, New York, USA), according to the manufacturer’s protocol. The primer sequences used were as follows: LARP1 sense 5′-GCAACCTAAAGACACTAC-3′ and antisense 5′-CCTCTTCTTCACTTCAATC-3′, PCNA sense 5′-GTACCTGAACTTCTTTACAAA-3′ and antisense 5′-TGCCTAAGATCCTTCTTC-3′, cyclinD1 sense 5′-GCTGCGAAGTGGAAACCA-3′ and antisense 5′-CCTCCTTCTGCACACATTTG-3′, and glyceraldehyde 3-phosphate dehydrogenase (GAPDH) sense 5′-GGAAGCTTGTCATCAATG-3′ and antisense 5′-CCCCACTTGATTTTGGAG-3′. The amplification conditions were as follows: 1 cycle of 2 min at 95 °C followed by 40 cycles of 10 s at 95 °C, 30 s at 60 °C, and 30 s at 72 °C. All experiments were performed in triplicate. The relative quantities of the expression of LARP1 were calculated by 2^−ΔΔCt^ (cycle threshold) values, with GAPDH as an internal control.

### Cell culture and plasmids

The CRC cells HCT8 and RKO were purchased from the Type Culture Collection of the Chinese Academy of Sciences (Shanghai, China). All the cells were cultured at 37 °C under a moist atmosphere with 5 % CO_2_ and maintained in Dulbecco’s modified Eagle medium supplemented with 10 % fetal bovine serum (Gibco, USA), 1 % streptomycin, and penicillin. The shRNA plasmid for LARP1 and the control-shRNA plasmid were purchased from Gene Pharma Company (Shanghai, China). For plasmid transfection, 4 × 10^4^ cells/well in six-well plates were cultured overnight and then transfected with plasmids using Lipofectamine 2000 (Invitrogen, CA, USA), following the manufacturer’s protocols. Stable clones of HCT8 and RKO cells expressing LARP1 shRNA or control shRNA were obtained by puromycin selection. The shRNA target sequence for LARP1 was 5′-GCCAGTCTCAGGAGATGAACA-3′. The control-shRNA target sequence was 5′-TGTTCATCTCCTGAGACTGGC-3′.

### Western blot analysis

Total protein was isolated using a radioimmunoprecipitation assay lysis buffer (Beyotime Biotechnology, China) according to the manufacturer’s instruction. Then, the concentration of extracted protein was measured using the bicinchoninic acid (BCA) protein assay kit (Beyotime Biotechnology). The same amount of protein (30 μg) was dropped into 10 % sodium dodecyl sulfate (SDS)-polyacrylamide gel for electrophoresis and then transferred onto polyvinylidene difluoride (PVDF) membranes. The membranes were blocked with 5 % fat-free milk in Tris-buffered saline and 0.1 % Tween 20 solution at room temperature, and then incubated with primary antibody against LARP1 (1:2000 dilution; Abcam, Cambridge, UK) and GAPDH (1:1000 dilution; Abcam, Cambridge, UK) at 4 °C overnight. Subsequently, the membranes were incubated with secondary antibody (1:5000 dilution; Abgent, San Diego, CA, USA), which has a role of horseradish peroxide (HRP)-tagged detection at room temperature for 2 h. Images of proteins were displayed using an enhanced chemiluminescence reagent (Applygen Technologies Inc., Beijing, China). The Quantity One software (Bio-Rad, California, USA) was used to quantitate the gray scale of each protein strips.

### Immunohistochemistry on tissue microarray

The TMA containing 117 paired CRC specimens was constructed in cooperation with Xin Chao Company (Shanghai, China). Tumors were resected from patients between May 2005 and March 2007. The follow-up lasted until December 2014. The median follow-up time in survivors was 63.5 months (range, 4–88 months). The TMA data include 55 men and 62 women with a mean age of 66.32 years (range, 26–89 years). After being dewaxed and rehydrated in xylene and a graded series of ethanol, antigen retrieval for tissue sections were implemented using 0.01 M sodium citrate buffer (pH 6.0). Then, the tissue sections were incubated in the dark with the primary antibody against LARP1 (1:500 dilution; Abcam, Cambridge, UK) or PCNA (1:10,000 dilution; Abcam, Cambridge, UK) for 16 h at 4 °C. The primary antibody was detected using an HRP-conjugated secondary antibody (Gene Tech, Shanghai, China) for 30 min at room temperature. The immunoreactivity was evaluated independently by two pathologists who were blind to the prognosis of patients. The evaluation included both the intensity and the area of staining. Staining intensity for LARP1 scores were as follows: 0 for negative, 1 for weak, 2 for moderate, and 3 for strong. The extent of staining was calculated according to the percentage of the cells stained positively; scores were as follows: 0 (0 %), 1 (1–25 %), 2 (26–50 %), 3 (51–75 %), and 4 (76–100 %). After multiplying the staining intensity score by the staining extent score, the specimens were divided into two groups based on the final score: low (0–6) and high (7–12). The PCNA index was based on the percentage points of the positive nuclear staining cells, and the specimens were divided into two groups based on this: low (less than 10 % of cells with positive nuclei) and high (more than 10 % of cells with positive nuclei).

### Cell proliferation assays

The cell proliferation ability was measured using Cell Counting Kit-8 (CCK8) (Dojindo, Japan) according to the instructions of the manufacturer. In simple terms, cells were seeded into 96-well plates (2 × 10^3^ cells/well) in triplicate. At the appropriate time (12, 24, 36, 48, 60, 72, 96 h), the cells were incubated with 10-μL CCK-8 solution for 2 h at 37 °C under a moist atmosphere with 5 % CO_2_. The absorbance of 450 nm was measured using the Gen5 microplate reader (BioTek, Vermont, USA).

### Plate colony formation assays

In the plate colony formation assays, 1000 log-phase cells per well were seeded in six-well plates and then cultured at 37 °C under a moist atmosphere with 5 % CO_2_ for 2 weeks. After fixing with methyl alcohol for 15 min, the cells were stained with Giemsa solution for 20 min. Then, counting and photographing were carried out for colonies of each well. All assays were repeated independently in triplicate.

### Statistical analysis

The SPSS statistical software program version 22 (SPSS, IL, USA) was used for statistical analysis. The two-tailed Student’s *t* test was used for continuous variables. The *χ* ^2^ test or Fisher’s exact test for categorical data was used to analyze the relationship between LARP1 and clinicopathological features. The disease-free survival (DFS) and overall survival (OS) rates were defined as the interval from the initial surgery to clinically or radiologically proven recurrence/metastasis and death, respectively. The Kaplan–Meier method was used to calculate the survival rates, and the differences between the survival curves were examined by the log-rank tests. Cox proportional hazards models were applied to estimate the effects of the expression of LARP1 and CRC clinicopathological variables on survival in univariate and multivariate analyses. *P* < 0.05 was considered statistically significant.

## Results

### Significant upregulation of the expression levels of LARP1 in human CRC

**Fig. 1:**
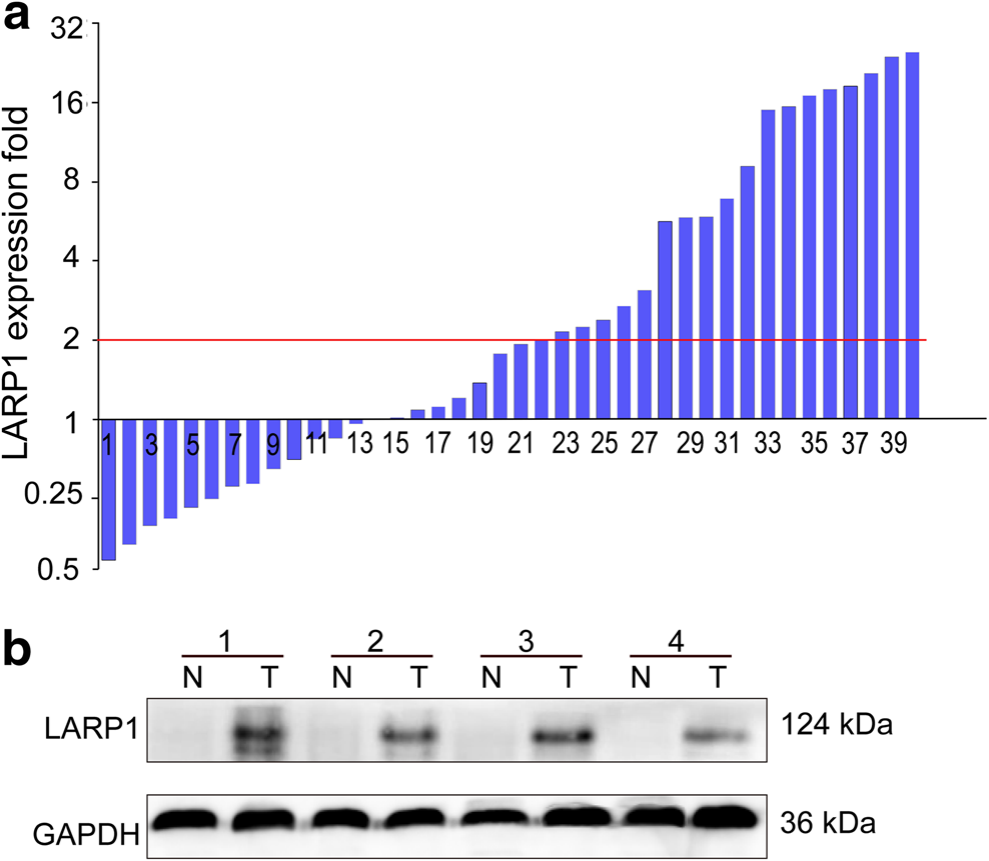
LARP1 expression in colorectal cancer tissues and adjacent normal mucosa. **a** Relative expression of LARP1 gene in 40 matched colorectal cancer tissue specimens compared with normal mucosa samples. The fold change of quantitative real-time polymerase chain reaction (RT-PCR) was calculated using the logarithmic scale of 2^−ΔΔCt^. **b** Western blot of LARP1 protein expression in representative four paired colorectal tumor tissues

The gene expression of LARP1 at the mRNA level was evaluated by the real-time PCR analysis of 40 pairs of colorectal cancer tissues and their paired adjacent normal mucosa. As shown in Fig. 1a, 19 (47.5 %) colorectal cancer tissues showed at least a twofold increase at the LARP1 mRNA level compared with the adjacent normal specimens. The mean relative quantification of LARP1 in the colon cancer tissue specimens (2.61 ± 0.55) was significantly higher than that in the normal tissue specimens (1.14 ± 0.23, *P* < 0.001 Student’s *t* test). The expression of LARP1 at the protein level was also confirmed using Western blot analysis. The LARP1 protein levels of tumor tissue specimens were significantly higher than those of the adjacent normal mucosa tissue (Fig. 1b). Thus, the expression of LARP1 was elevated at both transcriptional and posttranscriptional levels.

### Association of the expression of LARP1 and PCNA in colon cancer with clinicopathological factors

**Fig. 2:**
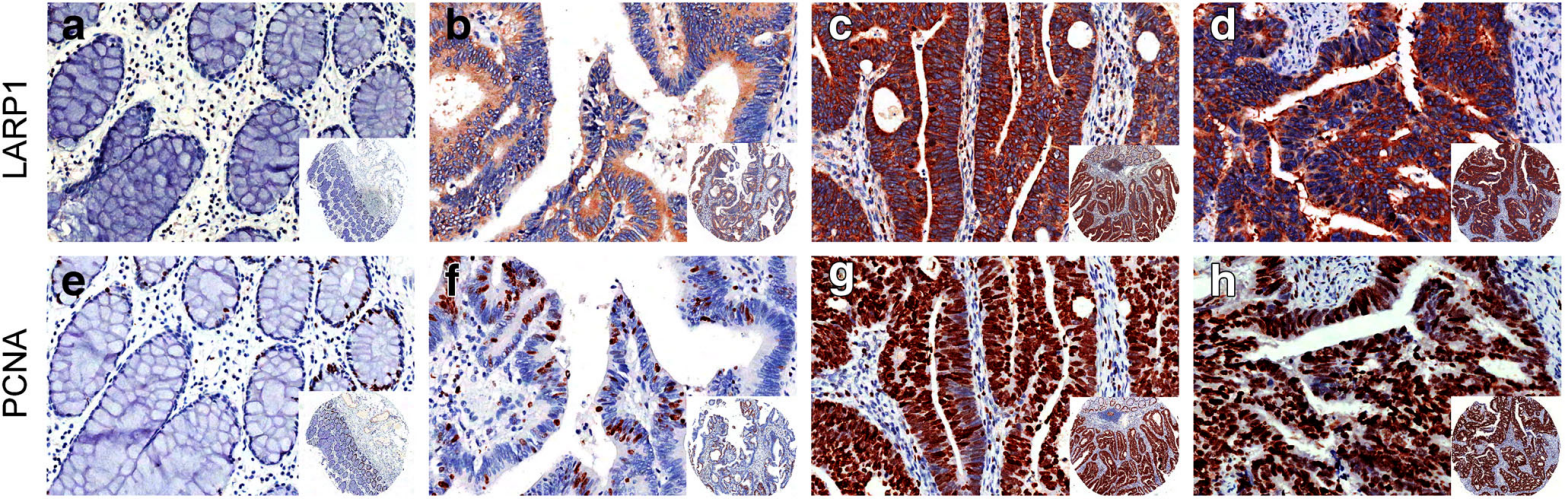
Immunohistochemical staining of LARP1 and PCNA expression in normal and colorectal cancer tissues. Representative images of LARP1 and PCNA expression in normal colonic epithelium (**a**, **e**), highly differentiated tumor tissues (**b**, **f**), moderately differentiated tumor tissues (**c**, **g**), and poorly differentiated cancer (**d**, **h**). The expression of LARP1 was higher in specimens with intensely positive PCNA staining. Original magnification ×200 (×40 for inset images)

Among the 117 CRC tissues and paired adjacent normal specimens on TMA, the immunohistochemical analysis of the expression of LARP1 and PCNA was performed. LARP1 was expressed in the cytoplasm rather than in the cell nucleus, while PCNA only expressed in the nucleus (Fig. 2). LARP1 showed various expression levels in colorectal tumor tissues, including weak, moderate, and strong. A conspicuous expression of LARP1 was apparent in 61 (52.1 %) of 117 colon cancer tissue specimens. The association between the expression of LARP1 and PCNA with clinicopathological factors is summarized in Table 1. The upregulated expression of LARP1 significantly correlated with T stage (*P* = 0.02), N stage (*P* = 0.006), M stage (*P* < 0.001), AJCC stage (*P* = 0.04), and differentiation rank (*P* < 0.001). The overexpression of PCNA was significantly associated with T stage (*P* = 0.012), N stage (*P* < 0.001), M stage (*P* = 0.01), and AJCC stage (*P* ≤ 0.001). Furthermore, the expression of LARP1 was higher in specimens in which PCNA staining was positive than those in which PCNA staining was negative. The positive correlation between LARP1 and PCNA in the area of proliferation was observed by using the Spearman’s correlation coefficient test (*r* = 0.332, *P* < 0.01) (Table 2).

**Table 1:** Association between clinicopathologic characteristics and LARP1 or PCNA protein expression

		LARP1		*P* value	PCNA		*P* value
		Low	High		Low	High	
Age (years)							
<65	50	25	25		21	29	
≥65	67	31	36	0.689	34	33	0.348
Gender							
Male	55	26	29		33	22	
Female	62	30	32	0.904	26	36	0.243
Location							
Left	64	33	31		38	26	
Others	53	23	30	0.379	18	35	0.378
T stage							
T1 + T2	23	16	7		18	5	
T3 + T4	94	40	54	0.02*	36	58	0.012*
N stage							
N0	64	38	26		44	20	
N1 + N2	53	18	35	0.006*	23	30	0.001*
M stage							
M0	102	55	47		49	53	
M1	15	1	14	0.001*	5	10	0.01*
AJCC stage							
I + II	63	33	30		40	23	
III + IV	54	20	34	0.04*	15	39	0.001*
Differentiation							
Well + moderate	98	54	44		50	48	
Poorly	19	2	17	0.000*	5	14	0.048*
Vessel invasion							
No	113	42	71		50	63	
Yes	4	2	2	1.000	0	4	1.000

*P* values are based on chi-square test or Fisher’s test

*AJCC* American Joint Committee on Cancer

*P < 0.05 was considered significant

**Table 2:** The association between LARP1 and PCNA protein expression

Tissue sample	PCNA expression		*P* value	*r*
	Negative	Positive		
LARP1 negative	36	20		
LARP1 positive	19	42	<0.001*	0.332

* *P* < 0.05 was considered significant

### Overexpression of LARP1 alone or combined with PCNA predicts poor prognosis

**Fig. 3:**
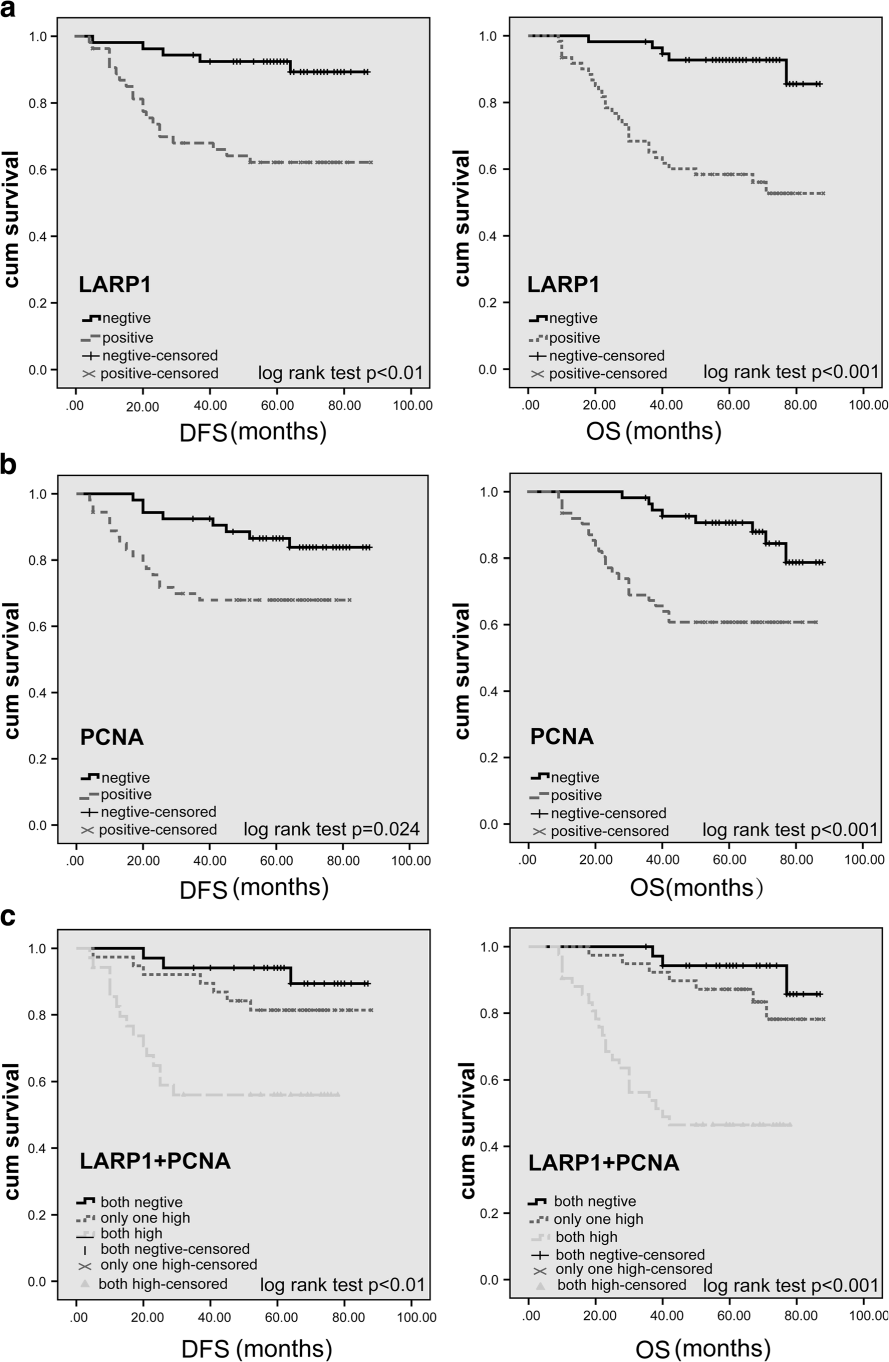
Kaplan–Meier survival analyses and log-rank test. **a** Disease-free survival (DFS) and overall survival (OS) of 117 patients correlated with LARP1 expression were determined by immunohistochemical staining of tissue microarrays. **b** DFS and OS were significantly higher in patients with PCNA-negative than in PCNA-positive tumors (*P* < 0.05). **c** DFS and OS were significantly lower in patients with tumors expressing high rather than low levels of both LARP1 and PCNA (*P* < 0.01)

The Kaplan–Meier analysis was used to show the relationship between patient survival (OS and DFS) and the expression of LARP1 or PCNA. The Kaplan–Meier plot showed that the patients with the elevated expression of LARP1 had a poorer OS (*P* < 0.001) and DFS (*P* < 0.01) than did the patients with LARP1-low tumors (Fig. 3a). The patients with an elevated expression of PCNA had a lower OS (*P* < 0.001) and DFS (*P* = 0.024) than did the patients with low PCNA expression (Fig. 3b). Furthermore, the samples were divided into the following three groups based on the concomitant expression of LARP1 and PCNA proteins: group 1, tumors exhibiting high expression of both proteins (LARP1+/PCNA+); group 2, tumors exhibiting high expression of only one protein (LARP1+/PCNA−, or LARP1−/PCNA+); and group 3, tumors exhibiting low expression of both proteins (LARP1−/PCNA−). Notably, a better OS and DFS were obtained in group 3 compared with group 2, while group 1 had the lowest OS and DFS compared with groups 2 and 3 (*P* < 0.01) (Fig. 3c).

A univariate analysis was performed using the Cox proportional hazards model. The result showed that tumor depth, AJCC stage, LNM stage, distant metastasis, vascular invasion, and expression of LARP1 and PCNA were associated with decreased OS (Table 3); all these characteristics, except tumor depth, were associated with decreased DFS (Table 4). The multivariate analysis demonstrated that the expression of LARP1 is an independent prognostic factor for OS (HR = 0.244; 95 % CI, 0.078–0.769; *P* = 0.016) and DFS (HR = 0.281; 95 % CI, 0.086–0.917; *P* = 0.035).

**Table 3:** Univariate and multivariate Cox proportional hazard models for overall survival (OS)

Variable	Univariate		Multivariate	
	HR (95 % CI)	*P* value	HR (95 % CI)	*P* value
LARP1				
Low	1		1	
High	0.154(0.059–0.401)	<0.001*	0.244(0.078–0.769)	0.016*
PCNA				
Low	1		1	
High	0.277(0.124–0.621)	0.002*	0.880(0.322–2.405)	0.804
Age				
<65	1			
≥65	0.804(0.393–1.646)	0.551		
Gender				
Male	1			
Female	0.616(0.300–1.262)	0.185		
Location				
Left	1			
Others	1.179(0.589–2.362)	0.642		
T stage				
T1 + T2	1		1	
T3 + T4	0.121(0.016–0.886)	0.038*	0.303(0.040–2.316)	0.250
N stage				
N0	1		1	
N1 + N2	0.105(0.040–0.272)	<0.001*	0.527(0.065–4.268)	0.548
M stage				
M0	1		1	
M1	0.024(0.010–0.059)	<0.001*	0.095(0.032–0.283)	<0.001*
AJCC stage				
I + II	1		1	
III + IV	0.082(0.029–0.234)	<0.001*	0.338(0.031–3.709)	0.375
Differentiation				
Well + moderate	1		1	
Poorly	0.134(0.066–0.273)	<0.001*	0.754(0.301–1.889)	0.546
Vessel invasion				
No	1		1	
Yes	0.230(0.070–0.758)	0.016*	0.208(0.052–0.826)	0.026*

*HR* hazard ratio; *CI* confidence interval

*P < 0.05 was considered significant

**Table 4:** Univariate and multivariate Cox proportional hazard models for disease free survival (DFS)

Variable	Univariate		Multivariate	
	HR (95 % CI)	*P*-value	HR (95 % CI)	P-value
LARP1				
Low	1		1	
High	0.210(0.079–0.560)	0.002^*^	0.281(0.086–0.917)	0.035^*^
PCNA				
Low	1		1	
High	0.395(0.170–0.916)	0.030^*^	0.960(0.353–2.612)	0.960
Age				
<65	1		1	
≥65	0.656(0.283–1.519)	0.325		
Gender				
Male	1			
Female	0.743(0.337–1.638)	0.462		
Location				
Left	1			
Others	0.905(0.406–2.014)	0.805		
T stage				
T1 + T2	1			
T3 + T4	0.148(0.020–1.097)	0.062		
N stage				
N0	1		1	
N1 + N2	0.101(0.035–0.296)	<0.001^*^	0.174(0.055–0.551)	0.003^*^
M stage				
M0	1		1	
M1	0.041(0.015–0.113)	<0.001^*^	0.145(0.038–0.543)	0.004^*^
AJCC stage				
I + II	1		1	
III + IV	0.119(0.039–0.334)	<0.001^*^	0.338(0.031–0.709)	0.003*
Differentiation				
Well + Moderate	1		1	
Poorly	0.177(0.078–0.405)	<0.001^*^	0.743(0.237–2.332)	0.611
Vessel invasion				
No	1		1	
Yes	0.219(0.051–0.937)	0.041^*^	0.169(0.032–0.903)	0.038^*^

HR: Hazard ratio; CI: Confidence interval

*P < 0.05 was considered significant

### Knockdown of the expression of LARP1 by shRNA inhibits CRC cell proliferation

**Fig. 4:**
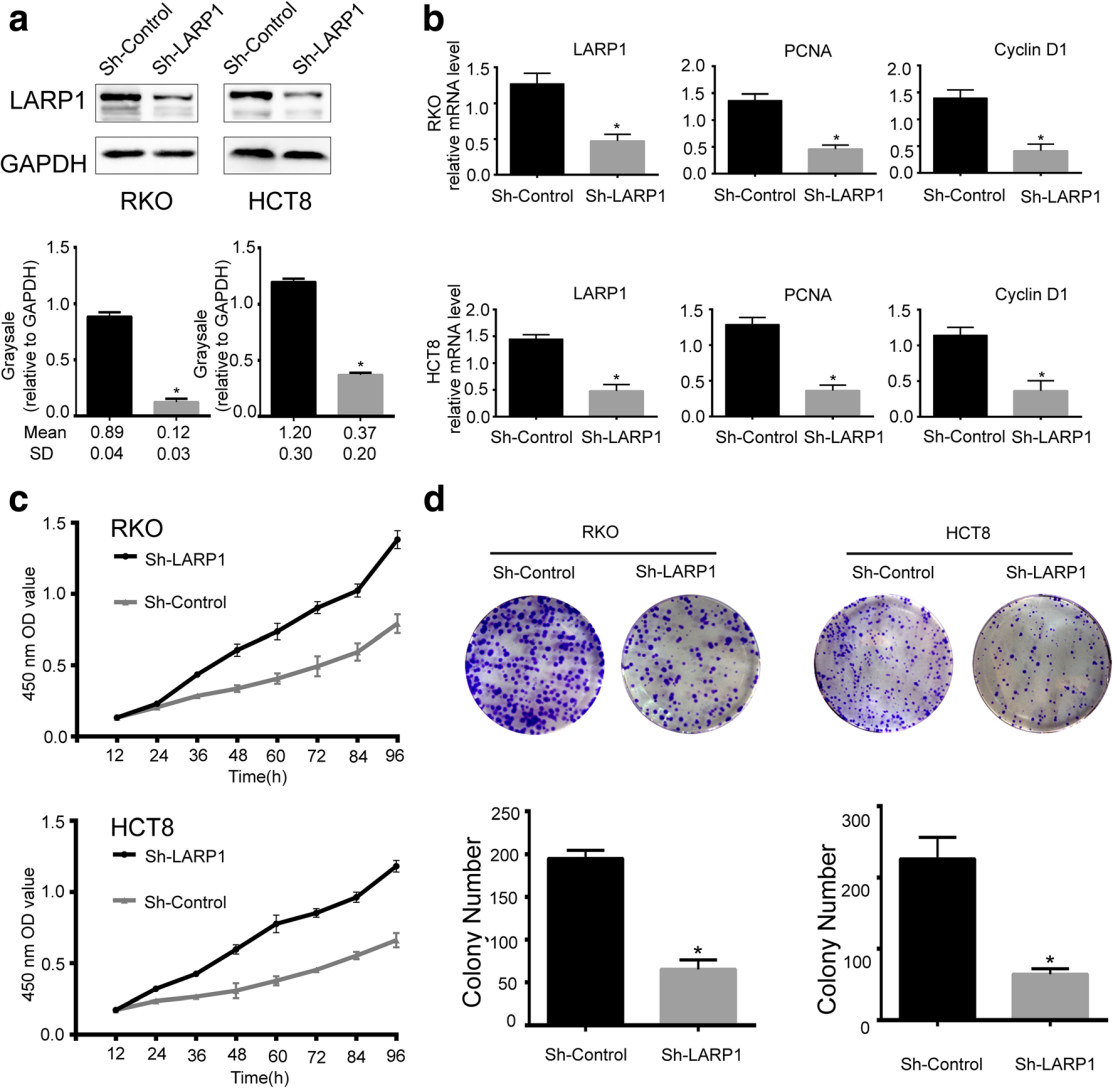
LARP1 knockdown inhibits colorectal cancer cell proliferation. **a** LARP1 level in stable knockdown HCT8 and RKO cell lines was assessed by Western blot. Grayscale values were analyzed using Quantity One software (*n* = 3, *P* < 0.05). **b** expression of proliferation-related genes was inhibited in LARP1 knockdown cells according to real-time PCR (n = 3; *P* < 0.05). **c**, **d** Effects of LARP1 knockdown on cell growth were evaluated by Cell Counting Kit-8 assays (**c**) and plate colony formation assays (**d**) (*n* = 3; *P* < 0.05)

To further determine the potential effects of LARP1 on CRC cell proliferation, the RKO and HCT8 cell lines were treated with LARP1-shRNA to knockdown the expression (Fig. 4a). To evaluate the effects of LARP1 knockdown on CRC cell proliferation, the quantitative PCR analysis was used to detect the expression of proliferation-related genes (PCNA; cyclin D1). The expression level of PCNA and cyclin D1 mRNA was downregulated in LARP1-shRNA cells (Fig. 4b). Then, CCK-8 and plate colony formation assays were used to estimate the role of LARP1 in CRC cell growth. As shown in Fig. 4c, the LARP1 knockdown cells were significantly reduced in cell proliferation compared with that of control shRNA cells. Furthermore, the ability of colony formation in the LARP1 knockdown cells were also reduced compared with that in control shRNA cells (*P* < 0.01) (Fig. 4d). These data showed that LARP1 contributed to CRC cell proliferation.

## Discussion

A great deal of evidence suggests that the LARP family is dysregulated in a variety of cancers [9]. LARP1 was the first member of the LARP family to be associated with oncogenesis [8]. It was first discovered in Drosophila. It plays a key role in embryogenesis, oogenesis, spermatogenesis, mitotic spindle formation, mitochondrial segregation, and cell cycle progression [24–26]. Although LARP1 plays an important role in tumorigenesis and progression of cervical cancer, non-small cell lung cancer, HCC, and prostate cancer [10, 18, 27, 28], the biological function and prognostic value of LARP1 expression in human colon cancer are still unknown.

This study identified the aberrant upregulation of LARP1 mRNA and protein in CRC tissues compared with paired normal specimens. Using immunohistochemical analysis, the upregulation of LARP1 in CRC tissues was found to be significantly associated with clinical pathological characteristics, such as tumor size, AJCC stage, depth of invasion, lymph node metastasis, distant metastasis, and tissue differentiation. The results demonstrated that LARP1 was a potential biomarker of colorectal cancer. As an important cell proliferation marker, PCNA plays an essential role in DNA replication and repair [20]. Its expression and distribution are closely associated with the rate of cellular proliferation and DNA synthesis [29]. This study found that the elevated expression of LARP1 correlated positively with PCNA expression, suggesting that LARP1 mediated cancer cell proliferation.

We carried out functional experiments to demonstrate the hypothesis that LARP1 promoted CRC cell proliferation. CCK-8 assays were used to illustrate the effect of LARP1 on CRC cell proliferation. The results showed that LARP1 knockdown in RKO and HCT8 cells inhibited cell proliferation. Using quantitative real-time PCR assays, we found that the expression of LARP1 and proliferation-related genes (PCNA; cyclin D1) was significantly downregulated in LARP1 knockdown cells. Furthermore, the colony formation ability was significantly decreased in LARP1-silenced cells. Our findings show that downregulation of LARP1 inhibits cell proliferation in CRC. It is well known that molecular targets play an important role in cancer therapy. The effect on cellular proliferation indicates that LARP1 is a desirable therapeutic target. Studies suggest that the phosphatidylinositol-3-kinase (PI3K)/AKT/mammalian target of rapamycin (mTOR) pathway promotes mRNA translation and increases protein synthesis, which affects both cell growth and proliferation [14, 19, 30]. It has been demonstrated that LARP1 expression correlates with poor prognosis of non-small cell lung cancer and promotes cellular proliferation by interacting with mTOR [10, 14, 19]. Additional studies are needed to investigate whether LARP1 mediated cellular proliferation and prognosis of colorectal cancer by interacting with mTOR.

To our knowledge, this study showed for the first time that overexpression of LARP1 alone or combined with PCNA predicts poor prognosis of patients with CRC. The Kaplan–Meier survival analysis showed that postoperative patients with high expression of LARP1 manifested poor OS *(P* < 0.001) and DFS (*P* < 0.01). In addition, the multivariate analysis using Cox proportional hazards model revealed that the expression of LARP1 is an independent prognostic factor for OS and DFS.

Nonetheless, this study also have some limitations. First, the interaction between LARP1 and mTOR in CRC cells was not validated in the present study. Second, the effects of LARP1 were not explored in vivo. Therefore, further studies are needed to confirm the hypothesis that LARP1 is a prognostic biomarker and a potential target for CRC therapy.

## References

[1] Siegel R, Naishadham D, Jemal A (2012). Cancer statistics, 2012. CA Cancer J Clin.

[2] Liu S (2015). Incidence and mortality of colorectal cancer in China, 2011. Chin J Cancer Res.

[3] Julie Bogaert HP (2014). Molecular genetics of colorectal cancer. Ann Gastroenterol.

[4] Prenen H, Vecchione L, Van Cutsem E (2013). Role of targeted agents in metastatic colorectal cancer. Target Oncol.

[5] Fleshman JW, Smallwood N (2015). Current concepts in rectal cancer. Clin Colon Rectal Surg.

[6] Akkad J, Bochum S, Martens UM (2015). Personalized treatment for colorectal cancer: novel developments and putative therapeutic strategies. Langenbeck’s Arch Surg.

[7] Fan J (2008). Gene-expression profiling in Chinese patients with colon cancer by coupling experimental and bioinformatic genome wide gene-expression analyses: identification and validation of IFITM3 as a biomarker of early colon carcinogenesis. Cancer.

[8] Stavraka C, Blagden S (2015). The La-related proteins, a family with connections to cancer. Biomolecules.

[9] Bousquet-Antonelli C, Deragon JM (2009). A comprehensive analysis of the La-motif protein superfamily. RNA.

[10] Liu D (2011). Activation of mammalian target of rapamycin pathway confers adverse outcome in non small cell lung carcinoma. Cancer.

[11] Wendel HG (2007). Dissecting eIF4E action in tumorigenesis. Genes Dev.

[12] Lee HW, Lee EH, et al. Prognostic significance of phosphorylated 4E-binding protein 1 in non-small cell lung cancer. Int J Clin Exp Pathol. 2015;8(4):3955–62. PMC4466968 26097581

[13] Castello A (2012). Insights into RNA biology from an atlas of mammalian mRNA-binding proteins. Cell.

[14] Mura M (2015). LARP1 post-transcriptionally regulates mTOR and contributes to cancer progression. Oncogene.

[15] Deragon J-M, Bousquet-Antonelli C. *The role of LARP1 in translation and beyond*. Wiley Interdisciplinary Reviews: RNA. 2015;6(4):399–417. 10.1002/wrna.1282 25892282

[16] Doerks T (2002). Systematic identification of novel protein domain families associated with nuclear functions. Genome Res.

[17] Aoki K (2013). LARP1 specifically recognizes the 3′ terminus of poly(a) mRNA. FEBS Lett.

[18] Xie C (2013). LARP1 predict the prognosis for early-stage and AFP-normal hepatocellular carcinoma. J Transl Med.

[19] Tcherkezian J (2014). Proteomic analysis of cap-dependent translation identifies LARP1 as a key regulator of 5’TOP mRNA translation. Genes Dev.

[20] Elias JM (1996). Cell proliferation indexed a biomarker in solid tumors. Biotechnic & Histochemistry.

[21] Guzinska-Ustymowicz K et al. Correlation between proliferation markers: PCNA, Ki-67, MCM-2 and antiapoptotic protein Bcl-2 in colorectal cancer. Anticancer Res. 2009;29:3049–52. 19661314

[22] Stoimenov I, Helleday T (2009). PCNA on the crossroad of cancer. Biochem Soc Trans.

[23] Zhang Z (2012). Structure of monoubiquitinated PCNA: implications for DNA polymerase switching and Okazaki fragment maturation. Cell Cycle.

[24] Sophie Chauvet CM-Z (2000). dlarp a new candidate Hox target in drosophila whose orthologue in mouse is expressed at sites of epithelium mesenchymal interactions. Dev Dyn.

[25] Ichihara K, Shimizu H, Taguchi O (2007). A Drosophila orthologue of larp protein family is required for multiple processes in male meiosis. Cell Struct Funct.

[26] Blagden SP (2009). Drosophila LARP associates with poly(a)-binding protein and is required for male fertility and syncytial embryo development. Dev Biol.

[27] Molinolo AA (2012). mTOR as a molecular target in HPV-associated oral and cervical squamous carcinomas. Clin Cancer Res.

[28] Kato M (2015). MicroRNA-26a/b directly regulate La-related protein 1 and inhibit cancer cell invasion in prostate cancer. Int J Oncol.

[29] Kato K (2011). Expression form of p53 and PCNA at the invasive front in oral squamous cell carcinoma: correlation with clinicopathological features and prognosis. J Oral Pathol Med.

[30] Laplante M, Sabatini DM. mTOR signaling at a glance. J Cell Sci. 2009;122(20):3589–94. 10.1242/jcs.051011 PMC2758797 19812304

